# Advances of endoscopic and surgical management in gastrointestinal stromal tumors

**DOI:** 10.3389/fsurg.2023.1092997

**Published:** 2023-04-12

**Authors:** Lei Yue, Yingchao Sun, Xinjie Wang, Weiling Hu

**Affiliations:** ^1^ Department of Gastroenterology, Sir Run Run Shaw Hospital, Medical School, Zhejiang University, Hangzhou, China; ^2^ Institute of Gastroenterology, Zhejiang University (IGZJU), Hangzhou, China; ^3^ Zhejiang University Cancer Center, Hangzhou, China

**Keywords:** gastrointestinal stromal tumors (GISTs), surgery, risk evaluation (RE), endoscopy, LECs

## Abstract

As one of the most common mesenchymal malignancies in the digestive system, gastrointestinal stromal tumors (GISTs) occur throughout the alimentary tract with diversified oncological characteristics. With the advent of the tyrosine kinase inhibitor era, the treatment regimens of patients with GISTs have been revolutionized and GISTs have become the paradigm of multidisciplinary therapy. However, surgery resection remains recognized as the potentially curative management for the radical resection and provided with favorable oncological outcomes. The existing available surgery algorithms in clinical practice primarily incorporate open procedure, and endoscopic and laparoscopic surgery together with combined operation techniques. The performance of various surgery methods often refers to the consideration of risk evaluation of recurrence and metastases; the degree of disease progression; size, location, and growth pattern of tumor; general conditions of selected patients; and indications and safety profile of various techniques. In the present review, we summarize the fundamental principle of surgery of GISTs based on risk assessment as well as tumor size, location, and degree of progress with an emphasis on the indications, strengths, and limitations of current surgery techniques.

## 1. Introduction

Gastrointestinal stromal tumors (GISTs) are rare mesenchymal subepithelial tumors in the alimentary canal with an estimated incidence worldwide of 1–2 per 100,000 (1, 2). As an oncological clinical entity, they were characterized as a virtually indolent property and could progress to highly aggressive malignancies (3). The anatomical position of GISTs nearly covers the complete gastrointestinal tract, primarily the stomach (50%–60%) followed by the small intestine (20%–30%), while they are infrequent in the colorectum and esophagus and rare in extragastrointestinal sites (mesentery and omentum) (4). GISTs are supposed to arise from the spindle-shaped interstitial cells of Cajal (ICCs) situated in the muscularis propria (MP) layer, known as pacemaker cells responsible for peristalsis, or their precursors (4, 5), with potential tumorigenesis referring to gain-of-function oncogenic activating mutation of receptor tyrosine protein kinase encoded by KIT or platelet-derived growth factor receptor alpha (*PDGFRA*) gene (6). Clinical manifestation of GISTs vary from asymptomatic incidental findings to palpable presentation including bleeding, obstruction, perforation, or abdominal mass (7). Histopathological biopsy and immunohistochemical analysis are of great significance to the diagnosis of GISTs (8). Therapeutic algorithms of GISTs incorporate surgical operation, endoscopy, interventional therapy, and medication, of which surgical resection of the entire tumor is considered to be the exclusive effective way to be completely curative for resectable primaries (9, 10). After the approval of imatinib mesylate by the Food and Drug Administration (FDA) of the United States in 2002 for molecular targeted medication, the availability of tyrosine kinase inhibitor (TKI) revolutionized the therapeutic management of GISTs, and GISTs have become the paradigm of multidisciplinary and multimodal therapy with reference to gastroenterologists, oncologists, and pathology specialists (11–13). Unfortunately, recurrence and metastasis remain common despite the remarkable efficacy of TKI therapy. (14). A growing number of investigations have demonstrated the safety and prognosis-improving benefits of surgery even in the metastatic scenario and updated original operative techniques (15). The present review aims to give an overview of updated surgical management in GISTs based on risk evaluation, progress degree, tumor size, and tumor location with introduction of modalities in the process of exploration.

## 2. Risk evaluation

The management modality of GISTs is dependent on preoperative risk evaluation with diversified regimens varying from procedures to chemotherapy being based on the location, size, and aggressiveness of GISTs. There are noteworthy limitations of the TNM staging system for assessing progression risk of GISTs, which is not regularly indicated in clinical practice (7). Tumor size and mitotic index were well-recognized prognosis indicators for resectable primaries of which tumor size in dimension <2 cm and mitotic count <5 per 50 high power fields (HPFs) are considered very low risk and tumor size in dimension >10 cm or mitotic count >10 per 50 HPFs are considered high risk in 2001 National Institutes of Health (NIH) taxonomies based on expert consensus (16). Considering patients with GISTs located in extragastric sites present with considerably higher risk of disease recurrence in comparison with those with gastric GISTs, another risk criterion for localized GISTs was developed by the Armed Forces Institute of Pathology (AFIP) with an additional incorporation of tumor location (Table 1) (17). Subsequently, modified NIH criteria, including mitotic count, tumor size, and location, especially tumor rupture, came into being as demanded under the situation where preoperative tumor rupture was found to be independently correlated with dismal recurrence-free survival (RFS) of GIST patients (Table 2) (18). Considering that tumor size and mitotic count are nonlinear continuous indices, precluding the accurate calibration of cutoff criteria, several prognostic contour maps, and nomogram, where these variables included in nonlinear modeling, have been proposed for the optimization of risk classification, which were validated to be more applicable for risk assessment of progressive aggravation (19, 20). Considering convenience and feasibility for clinical application in the Asian population, Chinese consensus guidelines for diagnosis and management of GISTs recommend modified NIH criteria for risk evaluation (21). However, above-mentioned AFIP criteria were suggested by the National Comprehensive Cancer Network of the United States (NCCN) and the European Society for Medical Oncology (ESMO) guidelines due to wide availability and prediction accuracy (22, 23). There are several limitations of existent risk classification criteria, which fail to perfectly predict metastasis and recurrence hazard for GISTs. Take SDH-deficient GISTs as an example, mitotic index was negatively associated with the risk of liver metastases but with a relatively extended period to develop metastases (21). Collectively, the establishment of new risk criteria-based prospective multicenter series is still warranted and the effective selection of multiple classifications for individualized patients under specific clinical circumstances is recommended for further formulation of treatment regimens.

**Table 1:** AFIP criteria for risk evaluation of metastasis or recurrence or tumor-related death for patients with primary GISTs (17).

Tumor size in maximal diameter (cm)	Mitotic count (per 50 HPFs)	Tumor location			
		Stomach	Jejunum and ileum	Duodenum	Rectum
≤2	≤5	0	0	0	0
>2 ≤ 5	≤5	1.9% (very low)	4.3% (low)	8.3% (low)	8.5% (low)
>5 ≤ 10	≤5	3.6% (low)	24% (moderate)	^a^	^a^
>10	≤5	12% (moderate)	52% (high)	34% (high)	57% (high)
≤2	>5	0	50% (high)	^a^	54% (high)
>2 ≤ 5	>5	16%( moderate)	73% (high)	50% (high)	52% (high)
>5 ≤ 10	>5	55% (high)	85% (high)	^a^	^a^
>10	>5	86% (high)	90% (high)	86% (high)	71% (high)

AFIP, Armed Forces Institute of Pathology; GISTs, gastrointestinal stromal tumors; HPFs, high power fields.

^a^ Sample numbers are too small to ascertain corresponding risk.

**Table 2:** Modified NIH criteria for risk evaluation of metastasis or recurrence or tumor-related death for patients with primary GISTs (18).

Risk stratification	Tumor size in maximal diameter (cm)	Mitotic count (per 50 HPFs)	Tumor location
Very low	≤2	≤5	Any
Low	>2 ≤ 5	≤5	Any
Moderate	>2 ≤ 5	>5	Gastric
	<5	6 ≤ 10	Any
	>5 ≤ 10	≤5	Gastric
High	Any	Any	Tumor rupture
	>10	Any	Any
	Any	>10	Any
	>5	>5	Any
	>2 ≤ 5	>5	Non-gastric
	>5 ≤ 10	≤5	Non-gastric

NIH, National Institutes of Health; GISTs, gastrointestinal stromal tumors; HPFs, high power fields.

## 3. Surgical management in gastrointestinal stromal tumors

### 3.1. Surgical management stratified by tumor stages

**Figure 1:**
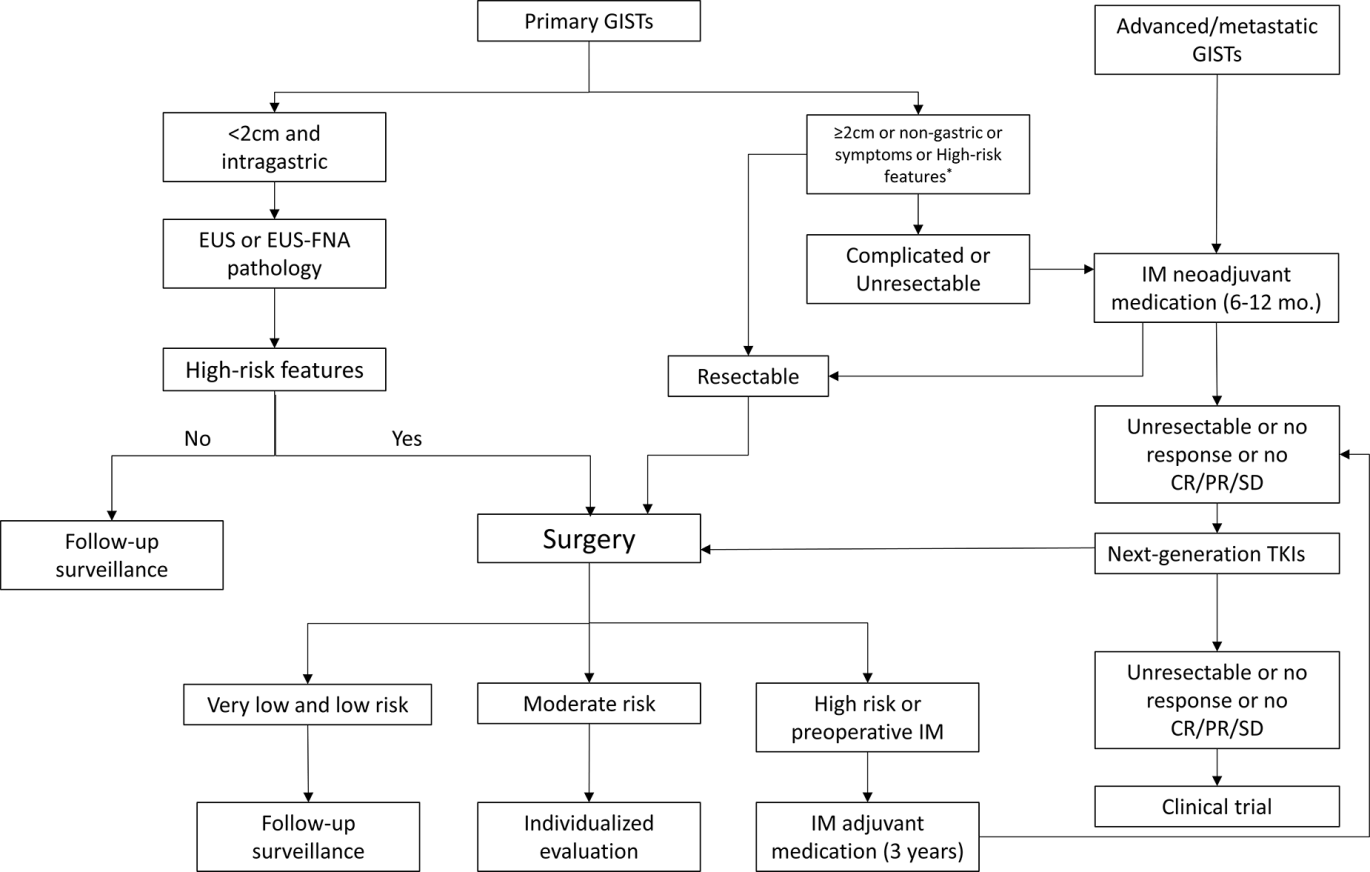
Management algorithm of primary and advanced/metastatic GISTs. GISTs, gastrointestinal stromal tumors; EUS, endoscopic ultrasonography; EUS-FNA, EUS-guided fine needle aspiration; IM, imatinib mesylate; TKIs, tyrosine kinase inhibitors; CR, Complete remission; PR, partial response; SD, stable disease; high-risk features refer to irregular and unclear border, echogenic foci, high mitotic rate, cystic degeneration, strong echo, ulcer, hemorrhage, and heterogeneity.

An overview of the management algorithm of primary and advanced/metastatic GISTs is summarized in Figure 1. The detailed surgical managements based on different tumor stages are as follows.

#### 3.1.1. Localized primary GISTs

Preoperative or intraoperative biopsy for pathological identification is not a prerequisite unless presurgical molecular diagnosis is necessary for targeted medication, which is not routinely recommended in cases with removable primaries to avoid the potential possibility of tumor rupture, bleeding, and diffusion (11). Among multiple biopsy approaches, endoscopic ultrasound-guided fine needle aspiration (EUS-FNA) is mostly used in clinical practice with distinct advantages of accuracy, safety, and relatively low possibility of dissemination. Potential limitations are inevitable considered that it is only applicable for GISTs situated in the lumen and fewer samples often lead to uncertainty of pathological diagnosis (24–26). Postoperative pathology report is of great value for the confirmation of diagnosis (27).

Surgery indications often refer to non-gastric GISTs, tumor size larger than or equal to 2 cm, or symptoms presented such as abdominalgia and gastrointestinal bleeding without regard to tumor size and location. Even though GISTs are located in the stomach with size less than 2 cm, attention should be paid on the risk under EUS or pathology for further determination of the implementation of operation. Higher risk tends to include irregular and unclear border, echogenic foci, high mitotic rate, cystic degeneration, strong echo, ulcer, hemorrhage, and heterogeneity (21, 28, 29). Without the presentation of these manifestations, regular follow-up surveillance is recommended.

It has been well-recognized that radical surgical resection is the first-line cornerstone and mainstay for the treatment of localized primary GISTs, which is the exclusive way for potentially thorough cure (30, 31). Surgery should be performed by experienced surgical specialists when open surgery or laparoscopy management was planned (7). The fundamental principle is to carefully complete *en bloc* removal of the tumor entity securing a sufficient histopathologically negative margin (R0 resection) achieving minor complications without damage of organ function and violation of tumor pseudocapsule prior to or during procedure as much as possible, which might contribute to accidental tumor perforation and rupture with tremendous risk of intraperitoneal implantation and spreading of tumor cells. To this end, “no touch, less compression” code and “extract bag” approach are available (21). Tumor excision incorporating peripheral tissues, whenever and wherever necessary and possible, in the case surrounding tissues or organs, is involved so as to realize the principle of R0 resection (7, 28). R0 resection is considered as the well-recognized favorable prognosis indicator for patients with localized primary GISTs (32). R1 resection (microscopically positive margins) is inclined to the development of tumor relapse, which occurs practically in all cases with tumor rupture (33). With respect to the situation where preoperative medication is unavailable or shows obscure benefits in patients with R0 resection that implicated major sequelae, R1 resection is recommended to be taken into consideration with no demonstration of correlation with dismal outcome especially in low-risk cases (23, 34). In addition, reoperation is a substitute for discussion as an individualized management after unexpected R1 resection and R2 excision (macroscopic incomplete excision) (7, 35). Inconsistent with surgical treatment of other malignancies, the proportion of lymphatic dissemination is comparatively diminutive and extensive lymphadenectomy is not regularly indicated with an exception of proof-positive lymphadenopathy, which implicated SDH-deficient genotyping especially in pediatric GISTs (27, 36, 37).

#### 3.1.2. Localized advanced GISTs

Preoperative neoadjuvant molecular targeted chemotherapy with TKIs like imatinib was indicated in situations where R0 resection is laborious to perform accompanied by alarmingly significant surgery risk and morbidity and increased possibility of perioperative complication like tumor rupture and bleeding or common postoperative functional sequelae, which will seriously affect organ function (23, 38). More often than not, potential occasion refers to large tumor size (>10 cm in maximal diameter) or difficult and unfavorable tumor sites located in operatively challenging areas such as the esophagus, esophagogastric junction (EGJ), duodenum, and rectum (39–41).

The rationale is that relatively large tumor size is reported to be positively correlated with the decreased rate of radical removal and increased risk of tumor recurrence (42). Consequently, shrinkage of tumor volume by neoadjuvant administration is of great significance for subsequent surgery, which not only contributes to improving the success rate of R0 resection and function preservation of involved organ and normal tissues to the greatest extent, as the avoidance of extended total gastrectomy and preservation of the anal sphincter, but also improve the 5-year overall survival (OS) and progression-free survival (PFS) (43–45). Simultaneously, genotyping tests prior to TKI therapy are essential for the identification of types of targeted drugs, and vigilant focus should be paid on imatinib-insensitive tumors such as PDGFRA D842 V mutations (46). Prior to the operation, neoadjuvant therapy should be conducted for 6–12 months so as to realize maximal chemotherapy outcome (29, 35). With respect to the embarking moment of surgery, the ESMO–EURACAN guidelines recommend to stop neoadjuvant therapy just before surgery and resume immediately after patients’ postoperative recovery (23). Further potent evidence based on large-scale multicenter clinical trials for the consolidation of efficaciousness of preoperative neoadjuvant medication remains to be developed.

Apart from aforementioned GISTs population with preoperative neoadjuvant chemotherapy with TKI, postoperative adjuvant therapy is often indicated as an indispensable part of standard treatment. Pertinent scenarios mainly refer to those categorized as “high risk” of recurrence and metastasis according to various classification criteria and those with R1 resection (11). According to a long-term randomized multicenter clinical trial, the postsurgical administration of TKI adjuvant chemotherapy was recommended for at least 3 years (47). Individualized evaluation is often recommended for moderate-risk patients to discuss whether to carry out adjuvant chemotherapy or follow-up surveillance (35).

#### 3.1.3. Recurrent, metastatic, or unresectable GISTs

Even though the tumor is completely removed through operation and TKI therapy presents amenable effectiveness, recurrence and metastasis are fairly commonplace, especially in high-risk patients with adverse 5-year OS and relapse-free survival (RFS) (48). Systemic therapy with TKI targeted medication based on genotyping is the first therapy and gold standard in recurrent, metastatic, or inoperable circumstances, and surgery is not generally recommended as a primary choice but considered as an available alternative and non-contraindication (28, 41, 49). As gross tumor volume (GTV) of GISTs is investigated to be positively correlated with tumor progression and negatively associated with the survival outcome of patients, debulking surgery and cytoreduction potentially decreased the likelihood of imatinib-resistant clones emerging, and the risk of secondary mutations has become a significant component of management in patients with recurrence or metastasis scenario (50, 51). Of note, debulking surgery is found to be efficacious in outcome improvement for most TKI-respondent patients and should be conducted before tumor progression (51, 52). Postoperatively, complete removal of tumor along with TKI therapy provides preferable outcomes for patients with more effectiveness and minimal complications and side effects compared with TKI alone (49, 53). As mentioned above, for those administrated with preprocedural TKI neoadjuvant regimens, adjuvant chemotherapy after surgery needs to be resumed as soon as possible (35). Specifically, patients who develop disease progression with TKI resistance will not benefit from identical TKI regimen used preoperatively, and postsurgical genetic test that is dedicated to identifying further therapeutic target is of vital value under this circumstance (41).

The indication of surgical administration in selected patients is as follows: better responding with imatinib therapy and tumor transfer from unresectable to resectable entity with minor operation-associated risk in residual lesions; localized progression with TKI resistance after targeted therapy including both sunitinib second-line therapy (54) and regorafenib third-line therapy (55) in addition to first-line treatment with imatinib (56); advanced tumor with a relatively small gross tumor volume of which lesion foci is evaluated to be completely removed through procedure; relatively limited and isolated metastatic foci or superficial lesions with operative feasibility; excision in cases with high risk of rupture necrotic metastases foci; patients in generally good condition and surgical tolerability; symptoms such as uncontrollable hemorrhage, perforation, and obstruction or acute pain with palliative emergency operation being necessary (28, 35). Surgical management for patients with general and disseminated progression following TKI medication is absolutely inadvisable, which could not present favorable survival benefits (21, 35).

Liver is one of the most common metastatic sites of GISTs; transcatheter arterial chemoembolization (TACE) and radiofrequency ablation (RFA) play a palliative adjunctive role in tumor control with feasibility and safety in addition to surgery (57–59). However, RFA is inclined to be suitable for tumors with a maximum diameter of 3 cm and is contraindicated in patients in whom tumor is contiguous with large vessels or supersedes liver capsule (7). Efforts has been made for the establishment of successful cases of liver transplantation but prospective research are still limited, and this management regimen is not regularly considered in clinical practice with scrupulous discussion processing required by multidisciplinary specialists for comprehensive prognosis evaluation of patients and conducted in experienced transplant centers (60, 61).

Taken together, multidisciplinary cooperation should be taken into consideration for the evaluation of feasibility of debulking operation and metastasectomy for patients with recurrent or metastatic GISTs and provide them with individualized treatment algorithm-integrated systemic medication with localized palliative procedure for further outcome improvement. If surgery is planned to be conducted, with the premise of ensuring R0 or R1 excision as much as possible, scrupulous consideration of patient selection refers to feasibility and complexity of operation, the degree of disease progression, the involvement of adjacent organs, the patient's age, physical condition, possible complications, postoperative recovery time, benefits, and risks.

### 3.2. Surgical management in tumor location subgroups

#### 3.2.1. Esophageal GISTs

Esophageal GISTs are comparably rare and ordinarily occur in the esophagogastric junction (62). Patients with relatively larger GISTs that are situated in the esophagogastric junction could benefit from TKI therapy followed by localized resection with avoidance of traumatic total gastrectomy, realizing organ function preservation to the greatest extent. Once the diagnosis of GISTs is established, surgery is suggested to be conducted immediately for such special anatomic location with operation difficulty (21). Choosing either complete esophagectomy or enucleation procedure is primarily based on the tumor size, location, and surgery-related risk (63). The enucleation technique could be a feasible alternative for relatively smaller and posterior wall GISTs, while radical esophagectomy is indicated for large tumors (4).

#### 3.2.2. Gastric GISTs

The gastric subgroup constitutes a majority of occurrence sites for GISTs. As mentioned above, on the premise of the absence of risk presentation under EUS, gastric GISTs with GTV of less than 2 cm without tumor-related symptom is indicated for follow-up surveillance. Surgery is recommended for cases inconsistent with these scenarios such as those in extragastric sites.

Due to the possibility of associated risk of local recurrences, enucleation is not generally recommended (35). However, in order to preserve normal organ function, enucleation approach could be tried with regard to small GISTs situated in the posterior wall (4). GISTs with a tumor size ≤4 cm located in the stomach are reported to benefit from resection by endoscopic techniques with adjuvant therapy or additional operation, when necessary, based on risk evaluation while superiority is not shown in cases with tumor size >4 cm in which surgery is often necessary (64). Surgical resection approaches of gastric GISTs primarily incorporate wedge resection (WR) or larger resection securing a margin of 1–2 cm (65). In the case where wedge resection is not feasible, segmental resection is an appropriate option referring Biliroth I, Biliroth II, and total gastrectomy with gastroduodenostomy, gastrojejunostomy, or Roux-en-Y reconstruction (4). Partial gastrectomy or total gastrectomy rather than proximal gastrectomy is recommended in GISTs located near the cardia or its extension (28, 35). Located in relatively favorable anatomic sites, greater curvature, or anterior wall of gastric body with tumor size of <5 cm is indicated for the application of laparoscopic procedure with safety, feasibility, less invasiveness, and comparable outcome compared to open surgery (21, 66, 67), and laparoscopic–endoscopic hybrid partial gastrectomy is a successful surgical method that secures complete resection without excessive violation of the normal anatomical structure (68). Simultaneously, this endoscopy-assisted laparoscopic operation is a recommendable alternative of open gastrectomy for gastric GISTs with feasibility and safety profile (69).

#### 3.2.3. Duodenum GISTs

Duodenum GISTs account for a practically small proportion of all GISTs with its second part, descending part, being the most preferred site (70). Operative procedure to completely remove tumor securing an adequate margin remains to be the optimal choice for radical cure in localized primaries with resectable feasibility. However, the complex and special anatomical position of the duodenum itself, which is contiguous with the head of pancreas, common bile duct, pancreatic duct, mesenteric blood vessels, and ampulla of Vater combined with relatively insufficient procedural experience owing to its rarity, brings about enormous challenge for performing the surgery (71, 72). The available operative approaches based on diverse instruments primarily consist of open surgery, endoscopy, laparoscopy, and hybrid surgery such as endo–laparoscopic cooperative procedure. Open surgery is applicable in most cases after feasibility and safety evaluation especially in ampulla of Vater and pancreas involved complex cases in which pancreaticoduodenectomy (PD), known as Whipple resection, is ordinarily necessary but with enormous invasiveness and damage to normal organ function (73, 74). Segmental intestinal resection can be performed when the tumor size is small and lesion is remote from the essential ampulla location. Followed by presurgical neoadjuvant TKI administration, limited resection instead of PD is often accessible, which decreases compromising the proximal structures due to large invasiveness. Limited resection approaches often incorporate WR and segmental duodenum resection with intestinal anastomosis according to the lesion site and extension (75). Endoscopic resection remains challenging and is intractable to securely perform with a comparable risk of nonradical resection and tumor rupture although there are advances in endoscopy techniques identically due to anatomic characteristics of the duodenum (76). In recent years, laparoscopy and endo–laparoscopic approaches combined hybrid surgery have been explored in the operative management of GISTs, with predominant advantages of minimal invasiveness, decreased perioperative complications, and long-term survival (77–80). Consequently, meticulous consideration should be taken referring to size, location, and invasiveness to adjacent structures for the individualized evaluation of optimal procedure option.

#### 3.2.4. Jejunum and ileum GISTs

The small intestine is the second most common site of gastrointestinal stromal tumors consisting of nearly a third of all GISTs (81). Open operation is ordinary practice while laparoscopic surgery should be taken into consideration for exploring and locating the corresponding lesion (21). In comparison with laparotomy, however, laparoscopic surgery is reported to assume less operation duration and less postsurgical complications as there was no significant between-group difference in oncological outcome according to a meta-analysis (82). Similarly, a retrospective review found that laparoscopic operation is safe and effective comparable to open operation in oncological prognosis and preferable in perioperative indicators (83). As for small intestine GISTs with size <10 cm in diameter, laparoscopic surgery can be recommended based on subgroup RFS analysis (84). Under normal circumstances, segmental resection with end-to-end intestinal anastomosis is recommended according to French clinical practice guidelines (35).

#### 3.2.5. Colon GISTs

Consistent with their incidence in the esophagus and the duodenum, GISTs situated in the colorectum are similarly rare. Compared with the left colon, GISTs tend to occur in the right side (85). All colon GISTs are recommended to be surgically resected regardless of tumor size and malignancy degree (86). Segmental resection with end anastomosis is the frequently used operative approach parallel with GISTs in the small intestine (35). Unsophisticated laparoscopic appendectomy is often applicable for particularly rare appendiceal GISTs (4).

#### 3.2.6. Rectum GISTs

GISTs located in rectum together with rectal vaginal space are strongly recommended for complete resection regardless of the tumor size since there are comparable risks for postsurgical recurrence and metastasis once diagnosis of GISTs is established (7) and enucleation is not regularly recommended (35). Considering that surgical difficulty and possibility of multivisceral resection will significantly increase with the enlargement of tumor size, operation should perform as early as possible (21).

There are different operative methods based on corresponding tumor location with respect to rectum GISTs. Low anterior resection and end-to-end intestinal anastomosis along with temporary colostomy are applicable for the upper section of the rectum with adequate distance to the anal sphincter. As for GISTs located in the lower rectum adjacent to the anal sphincter, abdominoperineal combined resection, known as Miles’ operation, with permanent sigmoid colostomy are commonly performed (4). Research reported that transanal full-thickness resection (FTR) presents more benefits concerning lower rectum GISTs and should be discussed when referring to minor tumor size (65). Original sphincter-sparing operation approaches with less invasiveness have been explored such as transanal endoscopic operation (TEO) (87) and transanal minimally invasive surgery (TAMIS) (88). Laparoscopic resection is another applicable option for lesions located in the upper rectum of <2 cm but not regularly recommended when the tumor increases in size (21). Preoperative neoadjuvant chemotherapy is necessary for the preservation of sphincter function as much as possible by tumor shrinkage with the improvement of outcomes (89). A multicenter cohort research performed by Wang et al. (90) was investigated to the compare oncologic outcomes in patients with low rectum GISTs between a local resection cohort and a radical resection cohort; they found that local resection presented a significant superiority in sphincter preservation, minimizing operation time. and postoperative complications compared to radical resection. However, in terms of tumor size >2 cm, there was a preferable survival outcome in the radical resection cohort.

#### 3.2.7. Extragastrointestinal GISTs

Actually, a great number of extragastrointestinal GISTs (EGISTs) reported are metastases foci of primary GISTs. Mesentery and omentum are common tumor sites of genuine primary EGISTs (91, 92). Other rare and unusual locations are pancreas (93), prostate (94), and pleura (95). With rare and aggressive malignancy, patients with EGISTs often have comparatively poor survival outcome, classified as high-risk lesions (96). Early identification and *en bloc* surgical excision as complete as possible remain the preferred treatment (97). Risk and cost-effectiveness of treatment regimens should be taken into account, and surgery combined with systemic neoadjuvant or adjuvant medication are often necessary (98) Cytoreductive debulking surgery might act as a potential palliative therapeutic strategy for symptomatic remission (99). Laparoscopy has been used for the surgical practice in EGISTs and achieved successful resection of tumor mass and oncological outcome as an available procedure option (100).

### 3.3. Surgical management in tumor size subgroups

GISTs with tumor size <2 cm are classified as small GISTs (101). More detailed subgroup categories include micro-GIST defined as GISTs <1 cm and mini GIST with size between 1 and 2 cm (102). Unless there are manifestations of high-risk characteristics based on EUS and biopsy, which warrant surgical management, the routine treatment for small GISTs is periodical follow-up surveillance with endoscopy and radiography (102). GISTs with size measuring equal to or larger than 2 cm (non-small GISTs) are recommended for surgical administration. With respect to those measuring over 5 cm accompanied with the presence or of symptoms or not, surgery is strongly indicated irrespective of whether the pathological diagnosis of GISTs is established or not (28).

Endoscopic resection is especially useful and safe for <2 cm GISTs. As for GISTs with 2–5 cm in size, enucleation, such as endoscopic submucosal dissection (ESD), is an available option ensuring complete excision but with risk of recurrence (28). As mentioned above, based on a long-term follow-up evaluation by Zhang et al., 4.0 cm might act as a threshold for choosing endoscopic resection in gastric GISTs stemmed from the MP layer with lower risk; however, surgery is still recommended for the population with increased risk (64). Laparoscopic resection also shows preferable outcomes and is recommended for GISTs between 2 and 5 cm, especially in easily accessible anatomic sites according to Chinese consensus guidelines (21, 103). A single-center long-term retrospective study was conducted for the comparison between surgery and endoscopy in the treatment of 2–5 cm GISTs. There were more complications and reoperation rates found in the endoscopic group compared to the surgery group with parallel outcome, and surgery, especially laparoscopic resection, is recommended more often (104). A size-matched comparison between laparoscopy and laparotomy found that gastric GISTs with size ≤8 cm might benefit more from laparoscopy based on oncologic outcomes (105). However, concerning relatively lager tumors (>5 cm) with excision needing larger incision, laparoscopic operation is not advocated due to pertinent tumor dissemination and open surgery is often encouraged (21). Patients with high risk, such as tumor size >10 cm in diameter or tumor size >5 cm plus mitotic count >5 per 50 HPFs, will benefit from neoadjuvant and adjuvant chemotherapy and achieve sound postsurgical oncological outcome.

## 4. Advances in laparoscopic operation

Under the premise of technical feasibility and the inexistence of operation contraindications, laparoscopy presents safe, efficient, and comparable postoperative oncological outcomes in selected patients with GISTs located in operationally facile sites like gastric and small bowel and with small size compared with open surgery (81, 106).

Recently, robotic surgical systems have been rapidly developed and received incremental interests. With kinetic stability, ergonomic design, and operation accuracy, they provide clinical surgeons with three-dimensional views and minimize the occurrence of tremors, thus reducing unnecessary tissue trauma as well as tumor manipulation and realizing the principle of minimally invasive surgery (107). Several reported research studies have confirmed the technical feasibility and safety of robot-assisted laparoscopic resection and suture reconstruction for the management of GISTs located in the upper gastrointestinal tract, especially for unfavorably positioned and relatively large series, which require more professional skills to avoid the risk of tumor rupture (108). Additionally, the Da Vinci Robot System has been introduced for optimization in laparoscopic operation, which is often preferred in complex and technically demanding cases (109–111). Researches of robot-assisted laparoscopic surgery are summarized in Table 3.

**Table 3:** Research studies of robot-assisted laparoscopic surgery.

Author	Year	Cases	Tumor location	Tumor size (cm)	Tumor pathology (no.)	Mean operation time (min)	Blood loss (ml)	Complete resection rate	Hospitalization duration (days)	Conversion to open surgery (no.)	Complication
Yamamoto et al. (112)	2021	1	Esophagus	4.3	GIST	319	135	100%	18	0	0
Grimaldi et al. (107)	2021	17	Stomach	6.0 ± 2.8	GIST	200 (105–313)	50 (50–100)	100%	5 (3–18)	0	0
Maggioni et al. (108)	2019	6	Stomach	6.3 ± 1.8	GIST	173 ± 39	NR	100%	3 ± 1	0	0
Dreifuss et al. (109)	2022	1	Stomach	2.3	GIST	82	NR	100%	2	0	0
Vicente et al. (113)	2016	6	Stomach (3), duodenum (3)	3.9 (2.4–5.5)	GIST	245 (150–540)	NR	100%	10.5 (6–24)	0	0
Hirata et al. (114)	2022	13	Stomach (12), duodenum (1)	3.5 (2.0–8.0)	GIST (12)	160 (82–270)	25 (5–50)	100%	3 (1–4)	0	0

NR, not reported; GIST, gastrointestinal stromal tumor.

## 5. Advances in endoscopy techniques

Different from gastrointestinal epithelial tumors, it is the occurrence site of GISTs that confine the application of endoscopy for the evaluation of tumor features and further resection. Of note, there are comparatively high perforation and incomplete excision rates during endoscopic operation especially in GISTs with larger size or extraluminal involvement (115). However, with the increasing maturity of endoscopy, several original endoscopic approaches have been investigated for the treatment of GISTs. According to research exploring conventional ESD for esophagus and stomach GISTs with 2–5 cm in size, the perforation rate was around 20%, and no recurrence or metastasis was observed (116). The high perforation rate remains challenging and fundus was identified as the risk location of complications (117, 118). Based on the consideration of limitation of conventional ESD, several modified techniques have been developed (Table 4). Endoscopic enucleation (EEN) is validated to be an effective method for resection (92.3%, 60 of 64) but without the avoidance of higher perforation in the fundus (119). Derived from ESD approach, the endoscopic muscularis dissection (EMD) procedure presents a sufficient complete resection rate for gastrointestinal mesenchymal tumors originating from the MP layer (120). Inconsistent with the circumferential incision in ESD, a longitudinal incision was performed followed by electrical or blunt dissection and clips closing. Although complete resection was achieved at 96.8%, there was a higher risk of perforation than that in ESD (121). Band ligation and resection (BLR) is another operation option of EEN assuming a comparably high resection rate (41 of 41) and nearly 10% (4 of 41) perforation (122). A modified ESD with enucleation was introduced for removing GISTs, and no serious complications were reported (123).

**Table 4:** Research studies of the conventional and modified ESD techniques.

Author	Year	Cases	Tumor location	Tumor size (cm)	Tumor pathology (no.)	Operation approach	Mean operation time (min)	Blood loss (ml)	Complete resection rate	Hospitalization duration (days)	Conversion to open surgery (no.)	Complication
Li et al. (118)	2013	11	Stomach	1.8 (1.2–3.0)	GIST (8)	ESD	81 (45–130)	NR	90.9%	NR	1%^a^	Perforation (27.2%)
He et al. (116)	2013	31	Esophagus (6) and stomach (25)	2.7 ± 0.7	GIST (31)	ESD	70.16 ± 16.25	NR	NR	NR	0	Perforation (19.35%) bleeding (9.68%)
Chen et al. (117)	2020	82	Stomach	3.5 ± 0.8	GIST (81)	ESD	68 (27–205)	NR	97.6%	16.1 (13.3–20.0)	0	Bleeding (9.8%) perforation (15.9%) infection (12.2%)
Jeong et al. (119)	2011	65	Stomach	1.4 (0.5–3.0)	GIST (26)	EEN	34.7 (6–90)	NR	92.3%	NR	NR	Perforation (12.3%)
Liu et al. (121)	2012	31	Esophagus (14) and stomach (17)	2.2 (0.5–4.5)	GIST (16)	EMD	76.8 (15–330)	NR	96.8%	NR	0	Perforation (12.9%)
Sawada et al. (120)	2020	6	Stomach	NR	GIMT	EMD	NR	NR	83%	NR	0	0
Ko et al. (122)	2019	41	Stomach	0.9 ± 0.5	GIST (17)	BLR	12 (4–52)	NR	100%	NR	0	Perforation (9.8%)
Chu et al. (123)	2012	16	Stomach	2.6 (2.0–4.2)	GIST (14)	modified ESD with enucleation	52 (30–120)	Minor	93.8%	NR	0	0

ESD, endoscopic submucosal dissection; EEN, endoscopic enucleation; EMD, endoscopic muscularis dissection; BLR, band ligation and resection; NR, not reported; GIST, gastrointestinal stromal tumor; GIMT, gastrointestinal mesenchymal tumor.

^a^ Conversion to laparoscopic wedge resection.

In order to preserve the integrity of the mucosa and avoid pertinent perforation, strictures, and scars induced by endoscopic procedure, endoscopic submucosal tunnel dissection (ESTD) (124), also known as submucosal tunnel endoscopic resection (STER) (125) or per oral endoscopic tumor resection (POETR) (126), has been introduced for the treatment of GISTs originating from the MP layer based on the peroral endoscopic myotomy (POEM) (127) approach (Table 5). Following the creation of mucosal entrance proximal to the tumor, approximately 5 cm, a tunnel between the submucosa and MP layer was developed and the tumor was completely removed. Endoclips were employed to seal off the entrance. ESTD is evaluated to be a curative treatment option for GISTs with less invasiveness and apparent postoperative complications (128). However, due to the instinct difficulty of tunnel development in thick stomach mucosa, the majority of ESTD were performed in the esophagus or esophagogastric junction with insufficient efficacy validation in the stomach (129, 130). Moreover, limited tunneling space will preclude the *en bloc* resection of large GISTs with the routine criterion of <4 cm (126, 130).

**Table 5:** Research studies of tunneling techniques.

Author	Year	Cases	Tumor location	Tumor size (cm)	Tumor pathology (no.)	Operation approach	Mean operation time (min)	Blood loss (ml)	Complete resection rate	Hospitalization duration (days)	Conversion to open surgery (no.)	Complication
Inoue et al. (124)	2012	9	Esophagus (3) and cardia (4)	1.9 (1.2–3.0)	GIST (1)	ESTD	152.4 (40–365)	NR	100%^a^	6.3 (3–16)	2	NR
Tan et al. (131)	2017	20	Stomach	1.8 ± 0.7	GIST (20)	STER	74.9 ± 32.1	NR	95%^b^	NR	0	Pneumoperitoneum (5.0%)
Chen et al. (132)	2017	180	Esophagus (124), EGJ (43), stomach (13)	2.6 (2.0–5.0)	GIST (28)	STER	45 (15–200)	NR	90.6%%^b^	3.2	0	8.3%
Chiu et al. (126)	2019	39	Esophagus (11), stomach (39), duodenum (11)	2.1 ± 1.4	GIST (15)	POETR	90.46 ± 46.49	NR	98.0%	3.2 ± 1.0	4	4.0%

ESTD, endoscopic submucosal tunnel dissection; STER, submucosal tunnel endoscopic resection; POETR, per oral endoscopic tumor resection; NR, not reported; EGJ, esophagogastric junction; GIST, gastrointestinal stromal tumor.

^a^ Of seven patients.

^b^ *En bloc* resection.

Endoscopic full-thickness resection (EFTR) cooperated with the full-thickness suturing technique will contribute to realizing radical resection of gastric GISTs located in the deep MP layer with endoscopically manageable complications and sound oncological outcomes, which is applicable to GISTs up to 4 cm and those located in all anatomical positions of the stomach (133). A dedicated full-thickness resection device for EFTR was applied in a 60-year-old patient with GIST with the advantage of protecting the peritoneal cavity from bowel contents (134). Another new technique called the clip-with-line traction-assisted preclosure assisted in EFTR was reported in a 47-year-old man with fundal GIST and was beneficial to preventing GIST falling into the abdominal cavity and the development of related complications such as peritonitis (135). Over-the-scope clip (OTSC) is an applicable device for the assistance of EFTR and verified with 100% excision success rate in treating GISTs with safety and effectiveness, which is especially recommended for a tumor size of <2 cm (136, 137). Researches of EFTR techniques are summarized in Table 6.

**Table 6:** Research studies of the EFTR technique.

Author	Year	Cases	Tumor location	Tumor size (cm)	Tumor pathology (no.)	Operation approach	Mean operation time (min)	Blood loss (ml)	Complete resection rate	Hospitalization duration (days)	Conversion to open surgery (no.)	Complication
Shichijo et al. (133)	2019	8	Stomach	2.0 (1.0–3.5)	GIST (8)	EFTR	67.5 (50–166)	NR	100%^a^	6 (4–11)	0	Perforation (63%)
Ren et al. (138)	2019	32	Duodenum	1.2 (0.5–3.0)	GIST (14)	EFTR	68 (17–186)	NR	100%^a^	6.2 (2–19)	1	Delayed perforation (3%)
Jung et al. (139)	2021	8	Stomach	1.6 (1.0–2.7)	GIST (8)	EFTR	66 (25–96)	NR	100%^a^	8.3 (5–18)	0	Bleeding (12.5%)
Wang et al. (136)	2022	40	Stomach	1.0 ± 0.3	GIST (36)	OTSC assisted EFTR	38.4 (13–157)	NR	92.5%	NR	0	0
Guo et al. (137)	2021	68	Stomach (64), duodenum (1), rectum (4)	1.3 ± 0.4	GIST (42)	OTSC assisted EFTR	53.7 ± 41.5	NR	98.5%	NR	0	Bleeding (1.5%), local peritonitis (2.9%)

EFTR, endoscopic full-thickness resection; OTSC, over-the-scope clip; NR, not reported; GIST, gastrointestinal stromal tumor.

^a^ *En bloc* resection.

## 6. Advances in laparoscopy–endoscopy cooperative techniques

Since endoscopic operation alone is faced with certain limitations, such as demanding skills of experienced endoscopists and high risk of procedural complications, the application of laparoscopy performed in the process of endoscopy plays a significant role in decreasing the perforation rate and improving the complete resection rate especially in relatively large tumors in size and has been expanded into clinical practice. Based on the roles of the two kinds of procedures in the operation process, operation modalities of these cooperative techniques primarily incorporate laparoscopy-assisted endoscopic surgery (LAES), endoscope-assisted laparoscopic surgery (EALS), and integrated laparoscopy–endoscopy cooperative surgery (LECS). With respect to LAES and EALS, one technique presents the basic role with the assistance of the other. However, laparoscopy and endoscopy teams cooperate with each other for the resection of lesion in LECS with essential significance rather than assistance.

### 6.1. Laparoscopy-assisted endoscopic surgery

Endoscopy plays a substantial role in LAES while laparoscopy provides backup and real-time control (Table 7). In the research by Qiu et al. (140), LAES, reported as laparoscopy-assisted endoscopic resection (LAER), was applied for resecting GISTs ≤3 cm in diameter. EMR or ESD was performed under endoscopy. The laparoscopy team facilitates the exposure and localization of the lesion from the perspective of peritoneal cavity. Through providing traction, lesions could be easily removed by endoscopy, especially with technical difficulty such as lesion located near the EGJ (141). Simultaneously, any complications such as perforation and bleeding that occur perioperatively could be treated immediately by laparoscopy. Controllable complications and no recurrence were observed. Apart from the stomach, lesions situated in the duodenum could also benefit from LAES with feasibility (142, 143).

**Table 7:** Research studies of the LAES technique.

Author	Year	Cases	Tumor location	Tumor size (cm)	Tumor pathology (no.)	Mean operation time (min)	Blood loss (ml)	Hospitalization duration (days)	Conversion to open surgery	Complication
Qiu et al. (140)	2013	5	Stomach	≤3	GIST	81.6 ± 31.8	29.8 ± 15.4	4.6	0	40%
Acker et al. (141)	2014	1	Stomach	3	Leiomyoma	NR	NR	NR	0	0
Kato et al. (142)	2011	1	Duodenum	2	GIST	200	Negligible	NR	0	Postoperative bleeding
Irino et al. (143)	2015	3	Duodenum	1.2–2.5	Adenocarcinoma	176–262	0–20	7–12	0	1/3

LAES, laparoscopy-assisted endoscopic surgery; NR, not reported; GIST, gastrointestinal stromal tumor.

### 6.2. Endoscope-assisted laparoscopic surgery

The lesion is removed by laparoscopic surgery with an endoscope contributing to the orientation and exposure of the mass. According to the various lesion locations and access approaches of laparoscopy, EALS mainly consists of endoscope-assisted wedge resection (EAWR), endoscope-assisted laparoscopic trans-gastric resection (EATR), and endoscope-assisted laparoscopic intragastric surgery (LIGS).

#### 6.2.1. Endoscope-assisted wedge resection

Table 8 summarizes corresponding researches of EAWR technique. With real-time monitoring, locating, and marking done by endoscopists, the lesion was removed by conventional laparoscopic wedge resection using a linear endoscopic gastrointestinal stapler. Subsequently, the mass resected was retrieved through the laparoscope followed by endoscopic examination of the existence of residual lesion and potential complications. EAWR is applicable for lesion not only in the anterior wall of the stomach but also in the posterior gastric wall, EGJ, and pyloric ring, which is more recommendable for an ultrasonic shear device or a vascular sealing system to avoid pertinent damage or stenosis (144, 145). More often than not, lesions situated in the posterior wall of the gastric body, limited to the central sections, were recommended for EAWR, which were easily accessible through the mentum or gastrocolic ligament by creating a small incision (146). In addition, the lesion with exophytic characteristic is another indication for EAWR.

**Table 8:** Research studies of the EAWR technique.

Author	Year	Cases	Tumor location	Tumor size (cm)	Tumor pathology (no.)	Mean operation time (min)	Blood loss (ml)	Hospitalization duration (days)	Conversion to open surgery (no.)	Complication
Kang et al. (144)	2013	101	Stomach	4.9 ± 0.6	GIST (78)	113 ± 36	36 ± 18	4.5 ± 2.1	0	2%
Wilhelm et al. (147)	2008	55	Stomach	2.54 (0.3–6.5)	GIST	81 (35–202)	NR	7.68 (4–19)	NR	NR
Qiu et al. (140)	2013	64	Stomach	3–5	GIST	86.3 ± 28.5	31.4 ± 11.6	3.5	0	40%
Kiyozaki et al. (148)	2014	42	Stomach	2–5	GIST	140 (89–307)	0	7 (6–14)	0	2.4%
Ismael et al. (145)	2016	1	Stomach	4.7	GIST	90	NR	3	0	0
Dávila et al. (149)	2016	38	Stomach	3.4 (0.7–7.5)	GIST (32)	106 (30–300)	NR	3	1	10.5%

EAWR, endoscope-assisted wedge resection; NR, not reported; GIST, gastrointestinal stromal tumor.

#### 6.2.2. Endoscope-assisted laparoscopic trans-gastric resection

EATR is more applicable for lesions located in the posterior gastric wall with a relatively large size or with intraluminal growth or near the EGJ (150, 151) (Table 9). As mentioned above, lesions located in the posterior gastric wall of the fundus or antrum instead of the body were preferred for this approach (146). With the assistance of endoscopic identification of the lesion location by palpation and diaphanoscopy, gastrotomy of the anterior gastric wall was performed by a laparoscopic team and the corresponding lesion was exposed. Subsequently, routine inverted laparoscopic wedge resection or full-thickness resection was conducted. Finally, the defect in the anterior gastric wall was closed using endoscopic stapler devices or laparoscopic sutures. Lesions located in the posterior of the duodenum were also indicated for applying EATR with robotic assistance. Based on the comparison analyses of Marzano et al., EAWR was recommended as the first choice for most gastric submucosal tumors (SMTs) than EATR due to gastrotomy and related complications such as increased operative time and blood loss, digestive fluid leakage, and the risk of abdominal cavity spread (152).

**Table 9:** Research studies of the EATR technique.

Author	Year	Cases	Tumor location	Tumor size (cm)	Tumor pathology (no.)	Mean operation time (min)	Blood loss (ml)	Hospitalization duration (days)	Conversion to open surgery (no.)	Complication
Wilhelm et al. (147)	2008	34	Stomach	2.61 (0.5–5.5)	GIST	114 (40–275)	NR	7.48 (2–14)	NR	NR
Sasaki et al. (150)	2010	10	Stomach	3.7 (2.2–5.0)	GIST (NR)	145 (100–240)	10 (3–65)	8 (5–9)	0	NR
Marzano et al. (152).	2011	1	Duodenum	3	Adenoma	270	200	7	0	0

EATR, endoscope-assisted laparoscopic trans-gastric resection; NR, not reported; GIST, gastrointestinal stromal tumor.

#### 6.2.3. Endoscope-assisted laparoscopic intragastric surgery

Endoscopy played a significant role in identifying lesion location, and then several laparoscopic trocars penetrated both the abdominal wall and the anterior stomach wall into the gastric cavity. Stay sutures could facilitate the lift of the anterior gastric wall to the abdominal wall. With the real-time monitoring and assistance of endoscopy during the operation, the lesion was resected through wedge resection or full-thickness resection by the laparoscopic team. Finally, the lesion specimen resected was retrieved perorally, and perforation and defect were closed by endoscopists and the laparoscopic team. Gastrotomy was performed when necessary, concerning large specimen with difficulty to be retrieved though the mouth (153, 154).

LIGS was first reported by Ohashi in 1995 and often recommended in lesions situated in the posterior gastric wall with intraluminal growth and relatively small tumor size (155, 156). Compared to EATR, LIGS is safer and with decreased blood loss and postsurgical complications through gastric perforation instead of gastrotomy.

In recent years, a modified LIGS technique, known as single-incision LIGS (sLIGS), has been reported by researchers. Only a 3 cm longitudinal incision was made near the umbilicus and a wound-protecting device was placed. Following a mini-size gastrotomy, three to four ports were placed through a single port device in the single incision. The lesion resection was similar with conventional LIGS and defect of gastrotomy and abdominal wall were closed. The indications for sLIGS were parallel to those of the routine LIGS technique. Of note, single incisions often correlated with higher perioperative security and decreased complications (157–160). Researches of LIGS technique are summarized in Table 10.

**Table 10:** Research studies of the LIGS technique.

Author		Year	Cases	Tumor location	Tumor size (cm)	Tumor pathology (no.)	Mean operation time (min)	Blood loss (ml)	Hospitalization duration (days)	Conversion to open surgery (no.)	Complication
Routine LIGS	Ludwig et al. (161)	2002	8	Stomach	NR	GIST (NR)	67.1 (49–102)	NR	10.2 (6–16)	1	12.5%
	Schubert et al. (162)	2005	7	Stomach	NR	GIST (NR)	83 (56–130)	NR	NR	NR	NR
	Privette et al. (154)	2008	4	Stomach	4.6 (2.5–7.5)	GIST (2)	236 (202–265)	100 (50–200)	3.3 (3–4)	0	0
	Dong et al. (163)	2013	6	Stomach	3.50 ± 0.84	GIST (6)	83.33 ± 26.58	<20	6.6 (5–7)	0	0
	Dong et al. (153)	2014	8	Stomach	2.75 ± 1.07	GIST (8)	85 ± 25.77	20 ± 10.35	7.5 ± 1.07	0	0
Single-incision LIGS	Na et al. (160)	2011	7	Stomach	2.7 (2.3–3.8)	GIST (5)	83.6 (70–105)	NR	5.4 (4–6)	0	0
	Vogelaere et al. (159)	2013	3	Stomach	3.8 (2.7–6.8)	GIST (3)	74.6 (67–82)	<30	5 (4–6)	0	0
	Katsuyama et al. (157)	2018	4	Stomach	3.0–4.3	GIST (2)	149 (116–170)	30 (0–43)	NR	0	25%
	Zhang et al. (158)	2022	13	Stomach	3.4 ± 0.2	GIST (10)	100 ± 10	50 ± 10	7 ± 1.5	0	8%

LIGS, endoscope-assisted laparoscopic intragastric surgery; NR, not reported; GIST, gastrointestinal stromal tumor.

### 6.3. Integrated LECS

#### 6.3.1. Classical LECS

LECS has been reported to successfully resect gastric submucosal GISTs, which is known as classical LECS (Table 11). First, ESD was performed *via* intraluminal endoscopy for circumferentially dissecting three quarters of the tumor submucosa. Next, the seromuscular layer was dissected by laparoscopy along the corresponding cut line, and the tumor was laparoscopically resected by a stapling device. There are several advantages of this technique, according to researchers’ opinion, such as it is independent of the tumor location without excessive sections of healthy tissues (164, 165). Theoretically, the application of the LECS technique is not restricted by the size of the tumor as the lesion is retrieved through the abdominal wall. However, LECS is shown to be more applicable for relatively small GISTs (166) and not recommended in large (> 5 cm) or ulcerative cases due to the increased risk of peritoneal contamination and tumor dissemination (167). Based on the research by Ri et al., subepithelial tumor located in the esophagogastric junction has a risk of conversing operation procedure from LECS to proximal gastrectomy, which is safer (168).

**Table 11:** Research studies of the classical LECS technique.

Author	Year	Cases	Tumor location	Tumor size (cm)	Tumor pathology (no.)	Mean operation time (min)	Blood loss (ml)	Hospitalization duration (days)	Conversion to open surgery	Complication rate (%)
Hiki et al. (165)	2008	7	Stomach	4.6 ± 0.3	GIST (6)	169 ± 17	7 ± 2	7.4 ± 8.1	0	0
Tsujimoto et al. (164)	2012	20	Stomach	3.8 ± 1.1	GIST (16)	157.5 ± 68.4	3.5 ± 6.4	11.6 ± 9.5	0	0
Hoteya et al. (169)	2014	25	Stomach	3.2 ± 1.4	GIST (16)	156.3 ± 50.5	NR	10.5 ± 2.4	0	0
Matsuda et al. (170)	2016	100	Stomach	3.1 ± 1.1	GIST (75)	174.3 ± 43.1	16.3 ± 37.5	8.4 ± 10.2	0	4
Obuchi et al. (171)	2014	1	EGJ	4.4	GIST	120	5	5	0	0
Ohi et al. (172)	2013	1	Duodenum	3.5	GIST	186	<10	5	0	0
Tsushimi et al. (173)	2014	1	Duodenum	0.8	NET G1	182	NR	9	0	0
Tamegai et al. (174)	2018	17	Colorectum	2.2 (0.8–4.1)	Adenoma (9)	183.3 (68–332)	7.8 (2–20)	6.4 (4–12)	0	0

LECS, laparoscopic and endoscopic cooperative surgery; NR, not reported; GIST, gastrointestinal stromal tumor; NET, neuroendocrine tumor; EGJ, esophagogastric junction.

#### 6.3.2. Laparoscopy-assisted endoscopic full-thickness resection

With the assistance of laparoscopy, the EFTR procedure achieved considerable improvement of avoiding excessive resection of normal tissues, and an original cooperative surgery based on the principles of LECS, called laparoscopy-assisted endoscopic full-thickness resection (LAEFR), has been developed by Abe et al. (175) (Table 12). Described as a hybrid natural orifice transluminal endoscopic surgery (NOTES), ESD followed by EFTR was endoscopically performed for the resection of 2/3 to 3/4 of tissue with the assistance of a laparoscopic team, which facilitates the exposure, and then the remaining tumor was completely resected and retrieved either perorally or through a laparoscope. The gastric wall defect was then hand-sewn and closed by laparoscopy. Apart from the accurate and complete removal of the tumor, the advantages of LAEFR incorporate minimal invasiveness, inexpensiveness in comparison with other laparoscopic surgery, and less perioperative adverse events managed *via* laparoscopic therapy. It is noteworthy that the closure of the artificial gastric wall perforation is easier and safer by means of laparoscopy compared to endoscopy (176). This technique is especially applicable for the resection of GISTs located in the MP layer with intraluminal growth modality (177).

**Table 12:** Research studies of the LAEFR technique.

Author	Year	Cases	Tumor location	Tumor size (cm)	Tumor pathology (no.)	Mean operation time (min)	Blood loss (ml)	Hospitalization duration (days)	Conversion to open surgery	Complication rate (%)
Abe et al. (175)	2009	4	Stomach	3.0 (2.2–4.3)	GIST (1)	201 (130–313)	27 (5–71)	7–8	0	0
Mori et al. (178)	2015	16	Stomach	28.3 (8–54)	GIST (16)	271	NR	12.3 (10–15)	0	NR
Lim et al. (179)	2017	8	Stomach	2.2 (2.1–3.3)	GIST (4)	127.5 (110–150)	30–100	4.5 (3–7)	0	0

LAEFR, laparoscopy-assisted endoscopic full-thickness resection; NR, not reported; GIST, gastrointestinal stromal tumor.

#### 6.3.3. Inverted LECS

In 2019, there was a case report in which a patient with remnant stomach GIST received inverted LECS for full-thickness resection with sound postoperative outcome (180) (Table 13). The brief operation modality of inverted LECS is described as follows. The first step is the identification of resection line and circumferential elevation of the gastric wall like a crown using stitches. Then, the seromuscular layer is dissected after artificial perforation followed by conducting EFTR. A .laparoscopic stapler is used to dissect the residual gastric wall, and tumor tissue is perorally retrieved. The final step is the defect suture by laparoscopic devices (181). In general, inverted LECS is performed with the traction inversion of tumor toward the intragastric cavity as a crown. There is, however, still relatively lower risk of gastric content spillage during this technique owing to gastric lumen exposure. Moreover, not all sites were feasible for inverted LECS such as the posterior wall.

**Table 13:** Research studies of the inverted LECS technique.

Author	Year	Cases	Tumor location	Tumor size (cm)	Tumor pathology (no.)	Mean operation time (min)	Blood loss (ml)	Hospitalization duration (days)	Conversion to open surgery	Complication rate (%)
Nunobe et al. (182)	2012	1	Stomach	6	Adenocarcinoma	152	0	NR	0	0
Aoki et al. (183)	2018	3	Stomach	11.7 ± 6.2	Carcinoma	192.3 ± 51.9	11.0 ± 6.5	17.0 ± 5.1	0	0
Takechi et al. (184)	2018	1	Stomach	5.8	Adenocarcinoma	215	0	NR	0	0

LECS, laparoscopic and endoscopic cooperative surgery; NR, not reported; GIST, gastrointestinal stromal tumor.

#### 6.3.4. “Nonexposure” LECS techniques

For the purpose of the reduction of tumor cells seeding into the abdominal cavity, several newer innovative “nonexposure” techniques, originated from the classical LECS procedure for full-thickness resection, named as a combination of laparoscopic and endoscopic approaches to neoplasia applying nonexposure technique (CLEAN-NET), nonexposed endoscopic wall-inversion surgery (NEWS), and closed-LECS, have been developed.

##### 6.3.4.1. Combination of laparoscopic and endoscopic approaches to neoplasia applying nonexposure technique

Inoue et al. took initiative to report the CLEAN-NET technique for the resection of a gastric neoplasm in 2012 (185). The corresponding procedures incorporate mucosal marking by endoscopy followed by the fixation of the mucosal layer into the seromuscular layer by four full-thickness stay sutures *via* laparoscopy. The next step is seromuscular layer dissection using a laparoscopic electrocautery knife and full-layer tumor dissection with pulling-out performed by a laparoscopic linear stapler, sealing the specimen into a protective mucosal “net.” It is obvious that the CLEAN-NET procedure is less invasive and has the capability of completely preventing between-luminal communication and thus consequent tumor dissemination and bacterial contamination. However, potential limitations refer to the risk of incomplete resection with positive margin, mucosal laceration, and incision line determination (80, 186, 187). To ensure the normal operation of the mucosal mechanical barrier and prevent mucosa tear, a tumor <3 cm in size is recommended for the application of this technique (181). Moreover, tumors located in technically demanding and inaccessible sites with the risk of deformity of the stomach, such as EGJ, the pyloric ring, the lesser curvature, and posterior wall, restrict the operation of CLEAN-NET due to potential risk of postoperative stenosis (188). A modified CLEAN-NET technique was introduced for the improvement of resection of gastric submucosal tumor especially in technically demanding locations and large cases (>3 cm), which secure the surgical field with anchor sutures and decrease stomach deformation and pertinent complications (189–191). Researches of conventional and modified CLEAN-NET technique are summarized in Table 14.

**Table 14:** Research studies of the CLEAN-NET technique.

Author		Year	Cases	Tumor location	Tumor size (cm)	Tumor pathology (no.)	Mean operation time (min)	Blood loss (ml)	Hospitalization duration (days)	Conversion to open surgery	Complication rate (%)
Original CLEAN-NET	Nabeshima et al. (187)	2015	2	Stomach	3.5–4.0	GIST (2)	165 (128–202)	16–29	8–9	0	NR
	Hajer et al. (192)	2018	2	Stomach	3.0–4.5	GIST (2)	150 (120–180)	<50	4–10	0	0
Modified CLEAN-NET	Hayase et al. (189)	2020	1	Stomach	3.7	GHIP	198	Minimal	9	0	0
	Fujishima et al. (190)	2017	13	Stomach	3.7 ± 0.9	GIST (13)	162 ± 59	7 ± 11	10 ± 7	0	8%
	Kanehira et al. (191)	2020	50	Stomach	3.5 (1.0–9.0)	GIST (25)	105.4 (50–220)	7.5 (1–50)	6–7	0	0

CLEAN-NET, combination of laparoscopic and endoscopic approaches to neoplasia applying nonexposure technique; NR, not reported; GIST, gastrointestinal stromal tumor; GHIP, gastric hamartomatous inverted polyp.

##### 6.3.4.2. Nonexposed endoscopic wall-inversion surgery

In 2011, the NEWS technique was first reported by Goto et al. (193) and developed as another novel nonexposure LECS, apart from CLEAN-NET, for full-thickness resection of gastric SMT without artificial perforation that avoids tumor seeding. Steps of NEWS primarily consist of marking mucosal and serosal surface around the tumor endoscopically and laparoscopically. Next, endoscopists inject sodium hyaluronate with an indigo carmine dye into the submucosal layer, which is beneficial to the following circumferential seromuscular dissection around the tumor by the laparoscopic team. Subsequently, tumor is inverted and suture closure of the seromuscular layer is performed laparoscopically. Finally, circumferential muco-submucosal incision of the intruded tumor is performed by endoscopists and tumor specimen is retrieved perorally followed by mucosal closure (80, 181, 194). Besides the benefit that precludes interluminal communication, NEWS could achieve an accurate determination of the resection line (195). However, it is obvious that the NEWS technique is restrained by tumor size as tumors with a relatively larger size (>3 cm) are laborious to be retrieved through the mouth. Simultaneously, tumor locations such as demanding EGJ and the pyloric ring also limit its application (181). NEWS is also found to be time-consuming; as reported by Mitsui et al., the median operation time in 28 patients with gastric GIST was 184 min (196). Researches of NEWS technique are summarized in Table 15.

**Table 15:** Research studies of the NEWS technique.

Author	Year	Cases	Tumor location	Tumor size (cm)	Tumor pathology (no.)	Mean operation time (min)	Blood loss (ml)	Hospitalization duration (days)	Conversion to open surgery	Complication rate (%)
Mitsui et al. (194)	2014	6	Stomach	2.3 (1.7–2.6)	GIST (5)	273 (140–397)	113 (0–250)	7–8	0	0
Kim et al. (195)	2016	1	Stomach	2.0	GIST	40	<50	10	0	0
Mahawongkajit et al. (197)	2017	1	Stomach	2.2	GIST	219	<10	5	0	0
Mitsui et al. (196)	2018	28	Stomach	2.4 (1.8–5.0)	GIST (28)	184 (98–357)	NR	nr	0	10.7%
Aoyama et al. (198)	2020	43	Stomach	24.6 ± 8.6	GIST (24)	198 (173–230)	5.0	7.0 (6.3– 8.0)	0	2.3%

NEWS, nonexposed endoscopic wall-inversion surgery; NR, not reported; GIST, gastrointestinal stromal tumor.

##### 6.3.4.3. Closed-LECS

Closed-LECS is also a completely non-open technique, which was reported by Kikuchi et al. for the resection of gastric SMTs (199). The detailed operation incorporates the following steps: routine ESD technique is performed after the submucosal injection. Subsequently, the incision line is marked by laparoscopy around the serosal surface followed by seromuscular suturing while inverting tumor into intragastric cavity. After the circumferential dissection of serosal muscular layer done by the endoscopic team, the lesion is removed perorally. Closed-LECS is more applicable for intraluminal and small GISTs with identical limitation of tumor size (<3 cm) for peroral approach (28). The operation process of the closed-LECS technique is roughly similar to NEWS with tumors all being retrieved perorally. Of note, endoscopic circumferential seromuscular incision is specifically performed in NEWS while not in closed-LECS (186). Researches of Closed-LECS technique are summarized in Table 16.

**Table 16:** Research studies of the closed-LECS technique.

Author	Year	Cases	Tumor location	Tumor size (cm)	Tumor pathology (no.)	Mean operation time (min)	Blood loss (ml)	Hospitalization duration (days)	Conversion to open surgery	Complication rate (%)
Kikuchi et al. (199)	2017	10	Stomach	2.4 ± 0.8	GIST	253 ± 45	18 ± 55	9.2 ± 1.5	0	10%
Saito et al. (200)	2020	3	Stomach	0.9 (0.7–1.4)	Adenocarcinoma	129 (115–148)	11 (3–15)	10 (7–15)	0	0

LECS, laparoscopic and endoscopic cooperative surgery; NR, not reported; GIST, gastrointestinal stromal tumor.

An overview and comparison of classical and modified LECS techniques are shown in Table 17. Collectively, a majority of newer innovated techniques are investigated in gastric neoplasm with little application reports in GISTs. The classical LECS technique will not cause mucosal defects and is independent of the size and location of the tumor, but there is a risk of abdominal spread. Modified LECS can prevent the tumor dissemination but is limited by the tumor size, location, and technical requirements. Individualized evaluation is necessary for selected patients for optimal operation approach. And further research studies in the GIST population are warranted to be investigated.

**Table 17:** Overview and comparison of integrated LECS techniques.

Characteristics	Classical LECS	Modified LECS				
		LAEFR	inverted LECS	CLEAN-NET	NEWS	Closed-LECS
Tumor location indication	Any	Any	Any	Anterior wall^a^	Anterior wall^a^	Anterior wall^a^
Tumor size indication	≤5^a^	≤5^a^	≤5^a^	≤3	≤3	≤3
Non-open procedure	No	No	No	Yes	Yes	Yes
First approach	Endoscopy	Endoscopy	Endoscopy	Laparoscopy	Laparoscopy	Endoscopy
Retrieval approach	Trans abdominal	Trans abdominal or transoral	Transoral	Trans abdominal	Transoral	Transoral
Suturing approach	Hand or linear stapler	Hand	Hand or linear stapler	Linear stapler	Hand	Hand

LECS, laparoscopic and endoscopic cooperative surgery; LAEFR, laparoscopy-assisted endoscopic full-thickness resection; CLEAN-NET, combination of laparoscopic and endoscopic approaches to neoplasia applying nonexposure technique; NEWS, nonexposed endoscopic wall-inversion surgery.

^a^ Recommended.

## 7. Conclusions

GISTs have been recognized as the paradigm of multidisciplinary and multimodal therapy integrating surgical resection with TKI neoadjuvant and adjuvant chemotherapy. Preoperative or postoperative molecularly targeted medication in a high-risk cohort could substantially contribute to the subsequent surgery resection and function preservation of the involved organ with improved survival outcome. Operation with complete resection is the mainstay for the management of patients with GISTs in clinical practice, which is often indicated in those with non-gastric GISTs, tumor size ≥2 cm, palpable symptoms, or EUS-related pathological risk. The advance of endoscopic and laparoscopic techniques, such as ESTD, EFTR, EAWR, and especially modified nonexposure LECS techniques have practically improved the success rate of operation, realized minimal invasiveness and more safety, and reduced perioperative complications. Robotic surgical systems are attractive treatment candidates for challenging cases. Tumor resection, to some extent, could be conducted and provide some earnings for properly selected patients with indications in recurrent and metastatic situation to improve their survival, but the benefits and risks should be considered comprehensively. Individualized evaluation from the multidisciplinary team and elaborative consideration of treatment algorithm for each patient are warranted. Further research studies in the GIST population are warranted.
